# Role of Neutrophil Apoptosis in the Resolution of Inflammation

**DOI:** 10.1100/tsw.2010.169

**Published:** 2010-09-01

**Authors:** Driss El Kebir, János G. Filep

**Affiliations:** Research Center Maisonneuve-Rosemont Hospital, Department of Pathology and Cell Biology, University of Montreal, QC, Canada

**Keywords:** neutrophils, apoptosis, programmed cell death, inflammation, resolution of inflammation, anti-inflammatory, mediators, intracellular signaling, lipoxins, cyclin-dependent kinase inhibitors, PDE4 inhibitors, NF-κB inhibitors, β2 integrin, formyl peptide receptors, airway inflammation, acute lung injury, sepsis, arthritis, ischemia/reperfusion, bacterial meningitis, trauma

## Abstract

Neutrophil granulocytes play a central role in host defense to infection and tissue injury. Their timely removal is essential for resolution of inflammation. Increasing evidence identified neutrophil apoptosis as an important control point in the development and resolution of inflammation. Delayed apoptosis and/or impaired clearance of neutrophils aggravate and prolong tissue injury. This review will focus on outside-in signals that provide survival cues for neutrophils, the hierarchy of pro- and antiapoptotic signals, and molecular targets in the antiapoptotic signaling network that can be exploited by endogenously produced bioactive lipids, such as lipoxins or pharmacological inhibitors, including cyclin-dependent kinase inhibitors, to redirect neutrophils to apoptosis in vivo, thus promoting resolution of inflammation.

## INTRODUCTION

Neutrophils or polymorphonuclear leukocytes play a central role in innate immunity by phagocytosing and destructing invading microorganisms through production of reactive oxygen intermediates and releasing proteolytic enzymes. Neutralization of the offending insult ideally prompts resolution of inflammation. However, this does not occur in many patients. Indeed, excessive or dysregulated neutrophil responses together with inadequate repair contribute to persisting tissue damage that underlies many inflammatory diseases. An important recent advance is the discovery that resolution of inflammation involves tightly controlled, active resolution programs[1,2]. During the past years, a number of novel cellular and molecular anti-inflammatory and proresolution pathways have been identified (for review, see [2]). A central paradigm has been that efficient resolution of inflammation depends on inhibition of neutrophil influx, promotion of monocyte recruitment, rapid clearance of infiltrating neutrophils, and regeneration of disrupted tissue structures[3,4]. The resolution interval or Ri (defined as the time required to reduce maximal inflammatory cell infiltration by 50%) has been proposed to define the kinetics of this process[2]. In addition to regulating recruitment and clearance, neutrophil apoptosis is another potential control point in resolving inflammation. Mature neutrophils undergo constitutive apoptosis in the circulation[5,6]. The life span of emigrated neutrophils can be influenced by signals from the inflammatory microenvironment. Prolongation of neutrophil life span is critical for their efficiency against infections[7]. Shortened neutrophil survival enhances susceptibility to recurrent infections in some pathological situations[8,9], whereas markedly delayed neutrophil apoptosis and/or decreased phagocytosis by macrophages may prolong and aggravate tissue injury[4,10,11]. This review will discuss how pro- and antiapoptotic signaling pathways may interact to determine the fate of neutrophils and ultimately the outcome of the inflammatory response. Information will also be provided on novel pharmacological strategies that could be used to facilitate the resolution of inflammation by redirecting neutrophils to apoptosis.

## MOLECULAR CONTROL OF NEUTROPHIL APOPTOSIS

### The Fate of Neutrophils

Neutrophils are terminally differentiated cells and have the shortest life span (8–20 h) among leukocytes in the circulation and die via a constitutively expressed cell death program. This mechanism is essential to keep the balance of cellular homeostasis under physiological conditions[5]. Apoptosis renders neutrophils unresponsive to extracellular stimuli and leads to expression of "eat-me" signals, so that neutrophils can be recognized and removed by scavenger macrophages[7,12]. Extravasated neutrophils may also be removed by recirculation back to the lymph nodes[13]; however, recirculation may not occur in the lung[14]. Neutrophil survival and death can be profoundly influenced by signals from the inflammatory microenvironment[5,6]. Proinflammatory mediators, including granulocyte macrophage colony-stimulating factor (GM-CSF) and IL-8, bacterial constituents, such as LPS and bacterial DNA containing unmethylated CpG motifs, or the acute-phase reactants modified C-reactive protein and serum amyloid A, could markedly prolong the longevity of neutrophils, whereas proapoptotic stimuli, such as TNF-α, TRAIL (TNF-related apoptosis-inducing ligand), or Fas ligand, shorten their life span[4,15,16,17,18,19]. Precise control of the neutrophil death program provides a balance between their defense functions and their safe clearance, whereas impaired regulation of neutrophil death is thought to contribute to a wide range of inflammatory pathologies.

### Multiple Pathways of Programmed Neutrophil Death

A complex network of intracellular death/survival signaling pathways regulates neutrophil apoptosis and the balance of these circuits would ultimately determine the fate of neutrophils. Apoptosis may result from activation of the extrinsic, intrinsic, or endoplasmic reticulum stress pathways. The intrinsic or mitochondrial pathway is likely initiated through ROS generation, although the mechanism(s) of ROS generation in nonactivated aging neutrophils is not clear. This is followed by loss of mitochondrial transmembrane potential ($\Delta\Psi_m$) and release of cytochrome c, apoptosis-inducing factor, and endonuclease G[5,6]. Cytochrome c induces the oligomerization of Apaf-1, which triggers the activation of caspase-9 and then caspase-3[20]. While IAP (inhibitor of apoptosis protein) family members can prolong cell survival through inhibiting both caspase-9 and -3[5], human neutrophils express IAPs at very low levels and their function in neutrophil apoptosis is uncertain. The extrinsic pathway triggers cell death following ligation of the cell surface death receptors, TNF-α or TRAIL receptors or Fas, to form the death-inducing signaling complex (DISC). Downstream signaling includes adaptor proteins, such as Fas-associated death domain (FADD), leading to cleavage of caspase-8[20]. The extrinsic pathway is not involved in constitutive neutrophil apoptosis, for neutrophils from Fas (*lpr)*-deficient mice undergo apoptosis at the same rate as wild-type mice[21] and neutralizing antibodies against Fas have no detectable effect on constitutive cell death[22]. Fas-mediated signaling overrides the antiapoptotic effects of GM-CSF through binding SHP-1[23]. Resistance to Fas-induced apoptosis may also contribute to delayed neutrophil apoptosis in patients[24]. It should be noted that the TNF-α exerts rather complex actions on neutrophils and could generate survival signals in neutrophils that were not initially killed by this cytokine[25]. Another mechanism that may regulate caspase-8 activity is translocation of cathepsin D from the azurophilic granules to the cytosol in a ROS-dependent and caspase-independent manner[26]. Pharmacological or genetic inhibition of cathepsin D results in delayed neutrophil apoptosis. Neutrophils also contain calpains, a family of noncaspase cysteine proteases, and calpastatin, a specific inhibitor of calpain-1[27]. In neutrophils undergoing apoptosis, calpastatin expression decreases, leading to increased activity of calpain-1, which cleaves and activates the proapoptotic factor Bax[28].

A role for autophagy in constitutive neutrophil apoptosis has been controversial. Autophagy-like cell death was observed in response to autoantibodies; however, other studies did not confirm this[5]. Recently, ROS-mediated neutrophil death distinct from apoptosis and necrosis has been suggested to be an essential step for the generation of neutrophil extracellular traps that bind and kill invading microorganisms[29].

## MAP Kinases

Multiple kinase pathways are involved in determining the fate of neutrophils. Activation of the MAPK/ERK and phosphoinositide-3-kinase (PI3K) pathways by proinflammatory mediators generates survival signals that inhibit the intrinsic pathway of apoptosis[5,6]. PI3K generates PtdIns(3,4,5)P3, which in turn activates Akt and influences NF-κB and cAMP-response-element-binding protein (CREB), thereby generating prosurvival signals. In addition, ERK 1/2 and Akt phosphorylate Bad and Bax, leading to dissociation of phosphorylated Bad and Bax from the antiapoptotic protein Mcl-1[17,30]. Concomitant activation of Akt and ERK is required for suppression of neutrophil apoptosis, and transient activation of Akt without ERK activation may not be sufficient to delay the death program. Contradictory results have been reported for p38 MAPK; its action on neutrophil survival may be stimulus and/or context specific (reviewed in [30]). For instance, the prosurvival function of p38 MAPK may include phosphorylation and, therefore, inactivation of caspase-3 and -8[31]. In other studies, constitutive neutrophil apoptosis was found to be associated with prolonged phosphorylation of p38 MAPK[17,18]. In sodium salicylate–treated neutrophils, activation of p38 MAPK results in reduction of Mcl-1 and acceleration of apoptotic cell death[32]. Caspase-3–mediated cleavage and activation of protein kinase C δ has also been implicated in constitutive neutrophil apoptosis[33], although the downstream targets of this kinase are largely unknown.

## NF-κB–Mediated Survival Signals

NF-κB controls transcription of a large number of proinflammatory genes as well as survival proteins that represents other signaling pathways for promoting neutrophil survival. Thus, pharmacological blockade of NF-κB evokes neutrophil apoptosis *in vitro* and could overcome the neutrophil survival effect conferred by LPS or TNF-α[34]. However, NF-κB inhibitors could also reduce release of survival factors from contaminating monocytes, which contributes to LPS-mediated neutrophil survival[35]. As NF-κB–regulated genes have also been implicated in the resolution of inflammation[36], timing of treatment with NF-κB inhibitors might be critical in order to avoid interfering with the successful resolution of inflammation *in vivo*.

## Mitochondria and Bcl-2 Family Proteins

Neutrophils express proapoptotic members of the Bcl-2 family, Bax, Bad, Bak, Bid, and Bik, as well as the antiapoptotic proteins Mcl-1, Bcl-$X_L$, and A1, but not Bcl-2[37]. Mcl-1 and A1 appear to be important in maintaining cytokine-regulated survival[38,39]. The cellular level of Mcl-1 rapidly decreases in neutrophils undergoing apoptosis, whereas prosurvival factors, such as GM-CSF and LPS, preserve Mcl-1 through inhibition of Mcl-1 degradation and induction of Mcl-1 gene transcription[40,41]. Cyclin-dependent kinases (CDKs)[42] and myeloid nuclear differentiation antigen (MNDA)[43] contribute to regulation of Mcl-1 turnover, although the precise molecular mechanisms remain to be investigated. A role for A1 is supported by the observation that *A1-a*$^{-/-}$ mice exhibited a higher rate of constitutive apoptosis in circulating neutrophils than wild-type mice[38]. Modulation of proapoptotic proteins also affects neutrophil apoptosis. For instance, Akt phosphorylates Bax at Ser184, thereby inhibiting Bax effects on the mitochondria through promoting heterodimerization with antiapoptic molecules Mcl-1, Bcl-$x_L$, or A1[44]. GM-CSF was found to induce Bim expression through activation of PI3K[45], challenging the previous concept of PI3K-mediated down-regulation of Bim expression[46]. Thus, induction of Bim gene expression by the prosurvival cytokines GM-CSF and G-CSF may activate a proapoptotic counter-regulation circuit[45].

Mitochondria play important roles in intracellular energy generation, apoptosis modulation, and intracellular redox signaling[47,48]. Mature neutrophils contain a low number of mitochondria that may have a role restricted to apoptosis[49]. Loss of $\Delta\Psi_m$ precedes development of apoptotic morphology in neutrophils undergoing constitutive[49] or induced apoptosis. Mitochondrial integrity is also regulated by $K^+$ efflux. Inhibition of this process prevents decreases in $\Delta\Psi_m$ and enhances neutrophil longevity by suppressing apoptosis[50].

## LIFE AND DEATH DECISIONS: HIERARCHY OF PROSURVIVAL AND PROAPOPTOTIC SIGNALS

The complex interactions between signaling pathways generating prosurvival and proapoptotic cues for neutrophils represent potential targets for therapeutic intervention. Since, under most conditions, neutrophils will be exposed to multiple mediators, their fate would ultimately depend on the balance between the pro- and antiapoptotic circuits. The following paragraphs provide examples of the hierarchy among these signals.

## Opposing Signaling through FPR2/ALX

Human neutrophils express the pleiotropic receptor formyl peptide receptor 2 (formyl-peptide-like 1 receptor/lipoxin receptor)[51]. This receptor binds a variety of ligands, including serum amyloid A (SAA), the anti-inflammatory lipids lipoxin $A_4$ ($LXA_4$) and aspirin-triggered 15-epi-$LXA_4$, annexin A1[52], and the antimicrobial cathelicidin peptide human CAP18/LL-37[53], and mediates opposing biological actions.

Annexin A1, a member of annexin family proteins that binds acidic phospholipids[54], is localized to the cytoplasm in unstimulated neutrophils. Following neutrophil activation, it is promptly translocated to the cell surface and secreted[55]. The N-terminal region of annexin A1 (protein Ac2-26) is responsible for accelerating neutrophil apoptosis[56]. Both annexin-1 and protein Ac2-26 inhibit neutrophil adhesion by blocking L-selectin shedding[56,57], thus generating a proapoptotic signal[58]. Caspase-3–mediated externalization of annexin A1 promotes clustering of phosphatidylserine receptors, ensuring efficient engulfment of apoptotic neutrophils[56].

The acute-phase reactant SAA possesses both beneficial and harmful actions in innate immunity[59]. SAA opsonizes Gram-negative bacteria and enhances phagocytosis, but it is also a precursor of amyloid

A, the deposit of which causes amyloidosis. SAA facilitates neutrophil and monocyte trafficking into inflammed tissues[51,60] and delays constitutive as well as Fas-triggered neutrophil apoptosis[17,61]. These actions are mediated via FPR2/ALX and involve ERK- and PI3K-mediated prevention of mitochondrial dysfunction and repression of caspase-3 activity[17,51]. The nucleotide receptor P2X7 has also been implicated in mediating SAA suppression of neutrophil apoptosis[61], although this has been questioned[62]. Like SAA, the bactericidal protein LL-37 also delays neutrophil apoptosis through evoking ERK phosphorylation and inhibition of caspase-3 activity[53]. This action may be advantageous for host defense against bacterial infections.

Other ligands of FPR2/ALX are $LXA_4$ and 15-epi-$LXA_4$ that are typically generated by transcellular biosynthesis at sites of inflammation (reviewed in [1]). Inhibition of cyclooxygenase-2 by aspirin or atorvastatin results in conversion of arachidonate to 15R-HETE that can be converted by neutrophils and other cells to 15-epi-$LXA_4$ and 15-epi-$LXB_4$[63,64]. $LXA_4$ and 15-epi-$LXA_4$ possess anti-inflammatory and proresolution actions. Lipoxins stimulate recruitment of monocytes, and inhibit neutrophil trafficking and accumulation in inflamed tissues (reviewed in [1,2]). Accumulation of $PGE_2$ at inflammatory sites induces a lipid mediator class switching from a predominantly 5-lipoxygenase activity to a 15-lipoxygenase activity generating $LXA_4$ parallel with the resolution of inflammation[65]. Thus, initiation of an inflammatory response would also activate subsequent proresolution mechanisms[66]. While lipoxins themselves do not appear to affect the apoptotic machinery, they facilitate nonphlogistic phagocytosis of apoptotic neutrophils[67,68]. *In vitro*, pretreatment or treatment with 15-epi-$LXA_4$ overrides the apoptosis-delaying effect of SAA and redirects neutrophils to apoptosis through attenuation of the prosurvival signals from SAA, leading to decreases in $\Delta\Psi_m$ and activation of caspase-3[17].

The molecular basis for how FPR2/ALX differently responds to various ligands remains to be explored. Direct competition of 15-epi-$LXA_4$ with SAA for binding to FPR2/ALX is unlikely because these ligands bind to distinct pockets on the receptor[60]. An intriguing possibility is that binding of 15-epi-$LXA_4$ may induce conformational changes in the receptor, thereby generating a yet unidentified negative signal that counters SAA signaling. Indeed, $LXA_4$ and15-epi-$LXA_4$ attenuate peroxynitrite signaling in human neutrophils[69], most likely via accumulation of presqualane diphosphate[70], which functions as an intracellular stop signal for neutrophil activation.

## Outside-in Signaling through $\beta_2$ Integrins

Integrins are heterodimeric transmembrane receptors, which mediate cell-cell adhesion and cell adhesion to the extracellular matrix[71]. In addition to mediating neutrophil migration[72] and phagocytosis[73], Mac-1 (CD11b/CD18) binding to its ligands fibrinogen[74], ICAM-1[75], or complement C3 fragment iC3b[76] also affects the neutrophil life span through integrating pro- and antiapoptotic signals[77]. ICAM-1 or fibrinogen induces Akt and ERK phosphorylation with subsequent suppression of neutrophil apoptosis. In the simultaneous presence of Fas ligand or TNF-α, the balance is, however, shifted towards cell death as a consequence of Akt inhibition by ROS-mediated activation of lyn and SHIP[77]. Mac-1–dependent phagocytosis of C3b-opsonized bacteria triggers ROS-dependent activation of caspase-8 and -3, thereby overriding the ERK-mediated survival signal, resulting in apoptosis[77]. Another ligand for Mac-1 is myeloperoxidase (MPO), the most abundant granule enzyme in neutrophils[78,79]. MPO, independent of its catalytic activity, rescues neutrophils from constitutive apoptosis through simultaneous activation of ERK 1/2 and Akt, Mcl-1 accumulation, and suppression of the mitochondrial pathway of apoptosis[62]. 15-epi-$LXA_4$ exerts multipronged actions to attenuate neutrophil responses to MPO. Down-regulation of Mac-1 expression and inhibition of neutrophil adhesion are important components of the anti-inflammatory activities of $LXA_4$ and 15-epi-$LXA_4$[1]. Induction of loss of Mcl-1 expression is likely the critical event in redirecting neutrophils to apoptosis by 15-epi-$LXA_4$. By attenuating up-regulation of Mac-1 expression and MPO release, 15-epi-$LXA_4$ interrupts the MPO-sustained autocrine/paracrine proinflammatory amplification circuit[80].

## NEUTROPHIL APOPTOSIS IN HUMAN DISEASE

Apoptosis is critical for the regulation of the life span of circulating as well as emigrated neutrophils. Execution of the death program can be either delayed or accelerated by signals from the inflammatory milieu. Suppressed neutrophil apoptosis or decreased uptake of apoptotic neutrophils by macrophages worsen damage to the host tissues[81] and impair wound healing[11]. Delaying neutrophil apoptosis exacerbates and prolongs inflammation[4,10] or even prevents spontaneous resolution of inflammation in experimental animals[82].

Suppressed neutrophil apoptosis has been detected in patients with inflammatory diseases, including acute respiratory distress syndrome (ARDS), pneumonia, sepsis, acute coronary artery disease, rheumatoid arthritis, and cystic fibrosis (Table 1). A frequent finding in these studies is the correlation of neutrophil apoptosis with the severity and/or outcome of the disease. Various mechanisms have been implicated in delaying neutrophil apoptosis in the blood, sputum, or synovial fluid. For example, enhanced GM-CSF production is thought to mediate suppression of neutrophil apoptosis in sepsis[83,84,85], severe burns[86], ARDS[87], or acute coronary artery disease[88], whereas activation of NF-κB–mediated survival signals may be involved in chronic obstructive pulmonary disease and respiratory syncytial virus infection–associated delay of neutrophil apoptosis[89,90]. Acute pancreatitis[24] and bacterial pneumonia[91] are associated with resistance to Fas ligand–induced apoptosis. In rheumatoid arthritis, impaired neutrophil apoptosis has been attributed to lactoferrin released from activated neutrophils[92] and SAA[61]. Multiple antiapoptotic signals generated during sepsis include release of pre–B-cell colony-enhancing factor[93], IL-10[84], and inhibition of translocation of MNDA from the nucleus to the cytoplasm[43], with subsequent inhibition of activation of caspase-3, -8, and -9, and preservation of Mcl-1. Acrolein, a toxic unsaturated aldehyde found in cigarette smoke prevents caspase-3 activation, presumably by direct inhibition of the enzyme[94].

Extending the life span of neutrophils at sites of infection is critical for optimal expression of their efficiency in eliminating pathogens[3,4]. Under certain conditions, shortening neutrophil longevity may comprise antimicrobial defenses and may contribute to susceptibility to recurrent severe infections (Table 1). Pyocyanin produced by the opportunistic pathogen *Pseudomonas aeruginosa* impairs neutrophil-mediated host defenses by attenuating neutrophil recruitment into the lung and accelerating neutrophil apoptosis[95]. Influenza virus A induces ROS-dependent[96], whereas HIV induces calpain-dependent, programmed death in neutrophils[8], culminating in neutropenia. Up-regulation of Fas is thought to contribute to accelerated neutrophil apoptosis in systemic lupus erythematosus and perhaps to autoantigen excess including dsDNA[97].

## NEUTROPHIL APOPTOSIS AS A THERAPEUTIC TARGET FOR ENHANCING THE RESOLUTION OF INFLAMMATION

New cellular and molecular circuits involved in the resolution of inflammation are being uncovered at a rapid pace. In some experimental models, a causal relationship has been detected between neutrophil apoptosis and outcome of acute tissue injury. Apoptotic neutrophil death *in situ* has multiple proresolution actions. Obviously, apoptosis renders neutrophils unresponsive to agonists, and apoptotic neutrophils stop producing and releasing proinflammatory mediators. Apoptotic leukocytes can sequester cytokines[98,99] and phagocytosis of apoptotic cells induces macrophages to switch from a proinflammatory to a proresolution phenotype[100]. Of note, injection of apoptotic neutrophils protects mice against LPS-induced shock[99]. Recent studies identified several classes of molecules for therapeutic induction of apoptosis in neutrophils for enhancing the resolution of inflammation (Table 2).

**TABLE 1**
**Altered Neutrophil Apoptosis in Human Disease**

| Disease State | Source of Neutrophils | Mechanism(s) | Ref. |
|---|---|---|---|
| Increased neutrophil apoptosis | | | |
| Influenza A virus | Peripheral blood | ROS-dependent apoptosis | |
| | | Increased Fas and Fas ligand expression | [96] |
| HIV | Peripheral blood | Calpain-mediated apoptosis | [8] |
| Liver cirrhosis | Peripheral blood | Increased caspase-3 activity | [9] |
| SLE | Peripheral blood | Increased Fas expression | [97] |
| Suppressed neutrophil apoptosis | | | |
| ARDS | BAL fluid | Enhanced G-CSF/GM-CSF production | [87] |
| COPD | Sputum | Increased NF-κB activity in neutrophils | [89] |
| Cystic fibrosis | Sputum | (?) | [131,132] |
| | Peripheral blood | Resistance to TNF-induced apoptosis | [103,132] |
| Bacterial pneumonia | BAL fluid | Resistance to Fas-induced apoptosis (?) | [91] |
| Respiratory failure in newborn infants | BAL fluid | (?) | [129] |
| RSV infection | BAL fluid | Enhanced PI3K and NF-κB activity | |
| | | Increased Mcl-1 expression | [90] |
| Cigarette smoke | Peripheral blood | Direct activation of caspase-3 by toxic aldehydes | [94] |
| Sepsis | Peripheral blood | Enhanced G-CSF production | [83] |
| | | Decreased caspase-3 and -8 activity | [93] |
| | | Reduced caspase-9 activity | [85] |
| | | Enhanced IL-10 production | [84] |
| | | Preserved Mcl-1 expression | [43,130] |
| | | Impaired MNDA translocation to the cytoplasm | [43] |
| Acute pancreatitis | Peripheral blood | Resistance to Fas-induced apoptosis | |
| | | Decreased procaspase-3 expression | [24] |
| Coronary artery disease | Peripheral blood | Increased IFNγ, IL-1β, and GM-CSF production | [88] |
| Rheumatoid arthritis | Peripheral blood | Lactoferrin-mediated suppression of apoptosis | [92] |
| | Synovial fluid | SAA-mediated suppression of apoptosis | |
| Severe burns | Peripheral blood | Enhanced GM-CSF production | [86] |

Abbreviations: ARDS, acute respiratory distress syndrome; BAL fluid, bronchoalveolar lavage fluid; COPD, chronic obstructive pulmonary disease; MNDA, myeloid nuclear differentiation antigen; RSV, respiratory syncytial virus; SLE, systemic lupus erythematosus.

## Cyclin-Dependent Kinase Inhibitors

The CDK inhibitors R-roscovitine (Seliciclib or CYC202), NG75, and hymenialdisine induce apoptosis in inflammatory cells through accelerating degradation of Mcl-1[42]. There is evidence that human neutrophils do express CDK1, CDK2, and CDK5[42,101], although little is known about their function in terminally differentiated neutrophils. Administration of R-roscovitine at the peak of inflammation attenuates

**TABLE 2**
**Therapeutic Induction of Neutrophil Apoptosis for Resolving Inflammation**

| Disease or Model | Species | Compound | Effects | Ref. |
|---|---|---|---|---|
| Carregeenan-induced pleurisy | Mouse | R-roscovitine | Decreased lung PMNs and monocytes | |
| | | | Increased PMN apoptosis | |
| | | | Enhanced phagocytosis of apoptotic PMNs | [42] |
| | Rat | IκBα repressor | Reduced tissue inflammatory cells | |
| | | | Increased leukocyte apoptosis | [118] |
| Carragenan plus MPO-induced ALI | Mouse | 15-epi-$LXA_4$ | Decreased lung PMNs | |
| | | | Increased lung monocytes/macrophages | |
| | | | Enhanced phagocytosis of apoptotic PMNs | |
| | | | Enhanced resolution | [80] |
| *Escherichia coli*–induced ALI | Mouse | 15-epi-$LXA_4$ | Decreased lung PMNs | |
| | | | Enhanced phagocytosis of apoptotic PMNs | |
| | | | Increased survival rate | [80] |
| LPS-induced ALI | Mouse | Metformin | Prevented NF-κB activation | |
| | | Rotenone | Decreased lung PMNs | |
| | | | Reduced severity of lung injury | [47] |
| | Mouse | Nutlin-3a | Increased PMN p53 expression | |
| | | | Enhanced PMN apoptosis | |
| | | | Reduced severity of lung injury | [121] |
| | Mouse | Decoy Ab against FcγR | Reversed LPS suppression of apoptosis | |
| | | | Decreased lung injury score | [126] |
| | Rat | IL-10 | Reduced lung PMNs | |
| | | | Enhanced PMN apoptosis | [125] |
| LPS-induced pleurisy | Mouse | Rolipram | Reduced lung PMNs | |
| | | | Enhanced PMN apoptosis | |
| | | | Enhanced resolution of pleurisy | [123] |
| Bleomycin-induced lung injury | Mouse | R-roscovitine | Enhanced PMN apoptosis | |
| | | | Reduced tissue injury | [42] |
| Cystic fibrosis | Human | R-roscovitine | Restored suppressed PMN apoptosis *ex vivo* | [103] |
| Pneumococcal meningitis | Mouse | R-roscovitine | Induced PMN apoptosis | |
| | | | Alleviated brain damage | |
| | | | Enhanced functional recovery | [102] |
| Arthritis | Mouse | R-roscovitine | Improved clinical scores | [42] |
| Severe trauma | Human | Agonistic Fas Ab | Induced PMN apoptosis through Fas *ex vivo* | [124] |
| Subcutaneous sponge implant | Rat | NF-κB decoy oligonucleotide | Enhanced PMN apoptosis | |
| | | | Enhanced phagocytosis of apoptotic PMN | [117] |

Abbreviations: Ab, antibody; ALI, acute lung injury; PMNs, polymorphonuclear neutrophils.

leukocyte accumulation and accelerates the resolution of pleural inflammation[42]. These actions can be prevented by zVAD-fmk, indicating caspase-dependent inflammatory cell apoptosis. Consistent with decreased pleural inflammation, R-roscovitine also reduces production of proinflammatory chemokines[42]. In the bleomycin-induced lung injury model, R-roscovitine reduces the number of neutrophils, but not monocytes/macrophages in the bronchoalveolar lavage fluid, attenuates lung injury, and decreases the lethality of bleomycin[42]. Accelerated neutrophil apoptosis is presumably responsible for these actions of R-roscovitine. Likewise, R-roscovitine treatment reduces hemorrhagic brain tissue damage and enhances recovery, as evidenced by a better reversal of hypothermia and faster recurrence of motor activity following pneumococcal meningitis in mice, coinciding with increased numbers of apoptotic neutrophils in cerebrospinal fluid[102]. Thus, R-roscovitine may alleviate brain damage, a severe clinical problem in bacterial meningitis despite optimal antibiotic therapy. In a model of passively induced arthritis, R-roscovitine improves clinical score faster than vehicle[42]. *In vitro* culture of neutrophils from patients with cystic fibrosis with R-roscovitine restores impaired apoptosis to normal levels[103]. Since the selective CFTR inhibitor $CFTR_{Inh172}$ does not affect programmed cell death in neutrophils from healthy volunteers, dysregulation of neutrophil apoptosis is likely not simply a consequence of chronic infection, but might also be related to yet unidentified modulatory factors intrinsic to cystic fibrosis. It remains to be investigated whether R-roscovitine treatment could also restore impaired macrophage phagocytosis, one of the characteristic features of cystic fibrosis.

## Lipoxins and Resolvins

Down-regulation of Mac-1 expression and inhibition of neutrophil adhesion are key components of the anti-inflammatory activities of lipoxins[1]. Consistent with Mac-1 modulation of neutrophil longevity, 15-epi-$LXA_4$ administered at the peak of inflammation enhances resolution of carrageenan plus MPO-induced and *Escherichia coli* septicemia–associated acute lung injury in mice and improves the survival rate[80]. MPO-deficient mice exhibit lower pulmonary bacterial colonization, reduced lung injury, and greater survival following injection of live *E. coli*[104] and show reduced neutrophil accumulation and tissue injury during ischemia/reperfusion[105]. Absence of MPO-derived oxidants is consistent with reduced tissue damage. It is not known whether MPO deficiency could affect the longevity of neutrophils. 15-epi-$LXA_4$ inhibits vascular permeability and release of the proinflammatory cytokines, further supporting its anti-inflammatory role[80]. 15-epi-$LXA_4$ reduces pulmonary neutrophil accumulation with concomitant increases in the number of apoptotic neutrophils, and facilitates recruitment of monocytes/macrophages and phagocytosis of apoptotic neutrophils and other cells[80], consistent with tissue repair[67,68]. Of note, a single injection of 15-epi-$LXA_4$ was found to be sufficient to accelerate the resolution of inflammation, indicating that once 15-epi-$LXA_4$ overcame prosurvival signals, neutrophil apoptosis would progress even in the absence of 15-epi-$LXA_4$. It should be noted that aspirin or lovastatin reduction of acid aspiration–induced lung inflammation is, in part, mediated through stimulation of 15-epi-$LXA_4$[106,107], although the effect of lovastatin on neutrophil apoptosis remains to be investigated. *In vitro*, aspirin or sodium salicylate counteracts prolonged neutrophil survival by LPS, but not by GM-CSF[108]. This action involves inhibition of NF-κB activation, likely via inhibition of peroxynitrite signaling[69] and acceleration of Mcl-1 degradation[32,109]. Consistently, both aspirin and sodium salicylate increase the percentage of apoptotic neutrophils and enhance their phagocytosis in thioglycollate-induced peritonitis[108].

Resolvins, derived from the enzymatic modification of ω-3 polyunsaturated fatty acids, also exhibits potent anti-inflammatory and proresolution activities, predominantly through decreasing the production of proinflammatory cytokines and inhibition of neutrophil recruitment[1,110]. Resolvin E1 enhances the survival of mice subjected to acid aspiration or *E. coli*–evoked pneumonia[111] and zymosan-inflamed peritoneum[13]. Resolvin E1 also protects the rat heart against reperfusion injury[112]. Resolvin D2 reduces excessive neutrophil trafficking and bacterial burden, and promotes phagocytosis of apoptotic neutrophils in cecal ligation and puncture-induced peritonitis in mice[113]. Whether these beneficial actions also involve induction of apoptosis in emigrated neutrophils remains to be investigated. Recovery from lung injury involves regulatory T cells[114], raising the intriguing possibility whether the proresolution mediators produced during interactions between Tregs and lung cells might lead to production of some of the lipid mediators discussed above[115].

## NF-κB Inhibitors

NF-κB plays a pivotal role in the regulation of inflammatory gene expression and the potential anti-inflammatory actions of NF-κB blockers have been investigated extensively[116]. By contrast, limited information is available on their impact on the resolution of inflammation. In a rat model of chronic inflammation, injection of an oligonucleotide decoy to NF-κB enhanced neutrophil apoptosis and phagocytosis by macrophages[117]. Increased apoptosis correlates with increases in p53 or Bax expression and decreases in Bcl-2 protein expression. Phagocytosis of apoptotic neutrophils by macrophages associated with reduction in TNF-α levels and stimulation of transforming growth factor-β1 production[118], consistent with tissue repair. Likewise, systemic injection of a cell-permeable form of IκBα (Tat-srIκBα chimera) shortly before administration of carrageenan reduces leukocyte recruitment into the pleural cavity in rats[118]. Inflammatory cells recovered from the pleural cavity display elevated caspase-3 activity and apoptosis. Surprisingly, local administration of Tat-srIκBα produces only marginal reductions in neutrophil migration[118], suggesting that NF-κB inhibition results in different actions on unstimulated and activated neutrophils, and thus the route of administration can determine the degree of inhibition in this model. Of note, the therapeutic effectiveness of these compounds may be limited in pathologies where GM-CSF is a predominant mediator of suppression of neutrophil apoptosis[83,86,87] because this cytokine does not signal through NF-κB[108].

## Inhibitors of the Mitochondrial Respiratory Complex I

Inhibition of mitochondrial respiratory complex I with metformin or rotenone attenuates neutrophil activation and reduces the severity of lung injury evoked by LPS[47]. A possible mechanism for these actions may be through intracellular $H_2O_2$-mediated inhibition of IκB-α degradation and thus prevention of NF-κB activation[119]. Blockade of complex I has similar actions in LPS-stimulated neutrophils and macrophages, suggesting that this is a general anti-inflammatory mechanism. Intriguingly, rotenone does not appear to induce apoptosis in human[120] and mouse neutrophils[119].

## Pharmacological Stabilization of p53

Recent results suggest that p53 regulates NF-κB activity in inflammatory cells. Mice deficient of p53 are more susceptible than wild-type mice to LPS-induced acute lung injury[121]. Treatment with nutlin-3a, a specific inducer of p53 stabilization, reduces the severity of lung injury evoked by LPS[121]. These results suggest reciprocal regulation of the activities of NF-κB and p53 in neutrophils and macrophages. It has been suggested that the beneficial action of nutlin-3a and p53 activation may, in part, result from enhanced neutrophil apoptosis induced by p53.

## Phosphodiesterase 4 Inhibitors

Phosphodiesterase 4 (PDE4) inhibitors induce increases in intracellular cAMP and exert potent anti-inflammatory actions[122]. Systemic administration of rolipram diminishes neutrophil accumulation and promotes neutrophil apoptosis in LPS-induced pleurisy in mice[123]. Resolution of pleurisy is associated with inhibition of the PI3K/Akt pathway, decreased levels of Mcl-1, and can be prevented by the pan-caspase inhibitor zVAD-fmk. Of note, rolipram also prevents NF-κB activation in infiltrating inflammatory cells, but this pathway does not appear to be relevant to resolution, for inhibition of NF-κB with PDTC or SN-50 fails to affect neutrophil accumulation in the pleural cavity[123].

## Fas Stimulation

Neutrophils from severely injured patients express a high level of Mcl-1, consistent with the prevention of collapse of mitochondrial function and cell death[124]. These events can be overcome by *ex vivo* cross-linking of the Fas receptor with immobilized agonistic anti-Fas IgM[124]. Fas stimulation evokes mitochondrial dysfunction and accelerates caspase-mediated Mcl-1 turnover. These observations suggest that neutrophils remain sensitive towards activation of the extrinsic apoptosis pathway despite an impaired intrinsic pathway. While selective Fas stimulation in neutrophils may represent a novel therapeutic opportunity, it remains uncertain how this could be achieved without potentially deleterious actions on other cells *in vivo*.

## IL-10

In a rat model of LPS-induced lung injury, intratracheal instillation of the anti-inflammatory cytokine IL-10 does not alter the onset or the magnitude of the initial inflammatory response to LPS, whereas it accelerates clearance of neutrophils from the alveolar space presumably through prevention of LPS-stimulated increase in neutrophil survival[125]. Whether IL-10 could exert similar beneficial actions when administered therapeutically remains, however, to be investigated.

## Decoy Anti-Fcγ Receptor Antibodies

Soluble immune complexes bind to the Fcγ receptors CD64, CD32, or CD16, and prevent neutrophil apoptosis *in vitro*[18,126]. *In vivo*, soluble immune complexes exacerbate LPS-induced lung injury, probably through protecting extravasated neutrophils from apoptosis[126]. Conversely, blockade of CD64 or CD32 with decoy antibodies reverses this protection and decreases lung injury score[126], an indirect indicator of resolution of inflammation.

## Targeting Caspase-1

Intratracheal instillation of LPS into caspase-1–deficient mice is associated with delayed neutrophil apoptosis and prolonged inflammation[127]. These findings were unexpected, for caspase-1 is not a part of the apoptotic machinery; instead, it indirectly affects apoptosis through cleavage of IL-1β[128]. Interestingly, IL-1β production was also detected in caspase-1–deficient mice. Thus, caspase-1 may have dual functions in the regulation of neutrophil apoptosis; it is proapoptotic in unstimulated cells, whereas this function is reversed in neutrophils exposed to LPS. While caspase-1 deficiency does not affect neutrophil migration into the lung, it delays resolution of inflammation, presumably by delaying apoptosis of neutrophils that have migrated into the lung.

## CONCLUSIONS

Resolution biology is an emerging discipline with a plethora of questions. This is a rapidly growing area of research in which new cellular and molecular circuits are being identified at a rapid pace. There are now several lines of evidence supporting a critical role for neutrophil apoptosis in determining the outcome of inflammation. Thus, suppressed neutrophil apoptosis contributes to persisting inflammation, whereas induction of apoptosis in neutrophils exerts anti-inflammatory actions and enhances the resolution of inflammation (Fig. 1). Small molecules affecting prosurvival signaling pathways are, therefore, promising tools for therapeutic interventions. A number of endogenous mediators and synthetic

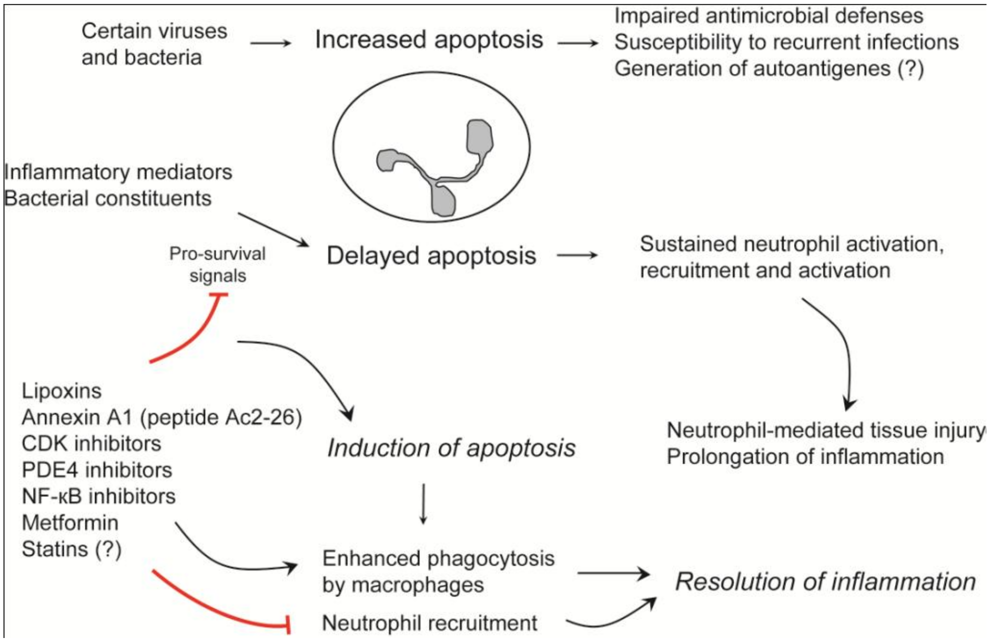

**FIGURE 1.** Neutrophil apoptosis influences the outcome of inflammation. Accelerated neutrophil apoptosis by certain viruses or bacteria increases susceptibility to severe recurrent infections and may contribute to generation of autoantigens. By contrast, suppression of constitutive neutrophil apoptosis by proinflammatory mediators or bacterial constituents leads to aggravated tissue injury and prolongation of the inflammatory response. Inhibition of neutrophil recruitment and redirecting neutrophils to apoptosis promote clearance of inflammatory cells and ultimately enhances the resolution of inflammation.

compounds has been described, which can redirect neutrophils to apoptosis and facilitate their clearance parallel with enhanced resolution of acute inflammation in experimental models. While clinical trials with these compounds remain distant, targeting neutrophil apoptosis holds promise as a disease-modifying intervention. Therapeutic efforts aimed at induction of neutrophil apoptosis at sites of inflammation may fulfill urgent, yet unmet, clinical needs to enhance resolution of inflammation.

## ACKNOWLEDGMENTS

This work was supported by grants MOP-64283 and MOP-67054 (to J.G.F.) from the Canadian Institutes of Health Research.
